# Identification of unrecognized host factors promoting HIV-1 latency

**DOI:** 10.1371/journal.ppat.1009055

**Published:** 2020-12-03

**Authors:** Zichong Li, Cyrus Hajian, Warner C. Greene

**Affiliations:** 1 Gladstone Center for HIV Cure Research, Gladstone Institute of Virology, San Francisco, California, United States of America; 2 Santa Rosa Junior College, Santa Rosa, California, United States of America; 3 Departments of Medicine and Microbiology and Immunology, University of California, San Francisco, San Francisco, California, United States of America; Vaccine Research Center, UNITED STATES

## Abstract

To counter HIV latency, it is important to develop a better understanding of the full range of host factors promoting latency. Their identification could suggest new strategies to reactivate latent proviruses and subsequently kill the host cells (“shock and kill”), or to permanently silence these latent proviruses (“block and lock”). We recently developed a screening strategy termed “Reiterative Enrichment and Authentication of CRISPRi Targets” (REACT) that can unambiguously identify host genes promoting HIV latency, even in the presence of high background “noise” produced by the stochastic nature of HIV reactivation. After applying this strategy in four cell lines displaying different levels of HIV inducibility, we identified FTSJ3, TMEM178A, NICN1 and the Integrator Complex as host genes promoting HIV latency. shRNA knockdown of these four repressive factors significantly enhances HIV expression in primary CD4 T cells, and active HIV infection is preferentially found in cells expressing lower levels of these four factors. Mechanistically, we found that downregulation of these newly identified host inhibitors stimulates different stages of RNA Polymerase II-mediated transcription of HIV-1. The identification and validation of these new host inhibitors provide insight into the novel mechanisms that maintain HIV latency even when cells are activated and undergo cell division.

## Author summary

The presence of a pool of latent HIV proviruses currently thwarts a cure for HIV-infected individuals. This “latent reservoir” is primarily composed of CD4 T cells that are infected with HIV but are indistinguishable from an uninfected T cell due to a lack of viral gene expression even when the cells are activated and undergo proliferation. This finding suggests there are powerful cellular mechanisms that hold HIV transcription in check even in stimulated cells allowing latent proviruses to remain hidden. Our goal was to identify and characterize these “unknown cellular factors”. We conducted genome-wide CRISPRi screens in multiple latently infected cell lines where each cell line displayed a different depth of latency as assessed by responsiveness to latency reversing agents. Application of our recently developed iterative screening strategy (REACT) allowed us to unambiguously identify and confirm four host factors that promote HIV latency. The latency promoting activity of these four factors (FTSJ3, TMEM178A, NICN1 and the Integrator Complex) were further validated in primary CD4 T cells, where their knockdown by shRNA significantly enhances latent HIV reactivation. In addition, we found that HIV infection preferentially occurs in cells expressing lower levels of these four factors. Mechanistically, our findings suggest that the newly identified host inhibitors likely block HIV transcription through different mechanisms. The identification and validation of these host inhibitors provides important new insights into how latency is maintained in T cells that could be useful for either activating and eliminating latently infected cells (“shock and kill”), or permanently silencing the integrated latent proviruses (“block and lock”).

## Introduction

Development of successful HIV cure strategies will be propelled by better understanding the mechanisms that maintain HIV-1 proviruses in a state of latency [1]. HIV latency may involve multiple mechanisms including repressive chromatin states [2,3,4,5], low levels of host transcription factors and Tat cofactors [6], transcriptional interference [7], and defects in RNA splicing and export [8]. Notably, most HIV proviruses from ART-suppressed individuals remain silent despite maximal T cell activation [9,10,11,12], and abundant evidence suggests that the mechanisms that maintain HIV latency remain intact even when cells are activated and undergo cell division [13,14,15,16,17,18].

Many HIV-1 cure-related studies have focused on the “shock and kill” approach that aims to re-activate latent HIV-1 with latency reversing agents (LRAs) and to eliminate the reactivated cells either through a viral cytopathic effect or as a result of immune cell-mediated clearance [19,20,21,22]. Mathematical modeling studies suggest that an “HIV cure” will require reduction of the size of the reservoir by >4 orders of magnitude [23]. To date, a variety of LRAs have been tested, including protein kinase C activators, histone deacetylase inhibitors, and bromodomain inhibitors [24]. Unfortunately, these agents only re-activate a small fraction of the pool of latently infected cells [10,25], and none have detectably reduced the size of the latent reservoir *in vivo* [26,27]. A more recent transcriptional silencing approach termed “block and lock” aims to permanently neutralize latent proviruses [28,29]. The Tat-inhibitor, didehydro-cortistatin A (dCA) [30], has shown some promise in this block and lock strategy. However, while delaying rebound, this small molecule does not completely prevent HIV-1 rebound [31,32]. Identifying the full set of host genes promoting HIV-1 latency could provide new and improved approaches for furthering both the “shock and kill” and “block and lock” therapeutic strategies.

To identify novel HIV-1 latency-promoting genes, we have recently developed a new screening strategy termed “Reiterative Enrichment and Authentication of CRISPRi Targets” (REACT) [33]. A major difficulty surrounding the screening for HIV-1 latency-promoting genes is the inherently stochastic nature of proviral expression [34,35]. As a result, the GFP-based HIV-1 latency models always display a small percentage of GFP-positive cells due to a low level of spontaneous virus expression that occurs in a stochastic manner [36,37]. This background noise could potentially obscure *bona fide* signals in a pooled genome-wide screen. REACT uses a catalytically dead Cas9 protein fused to the Kruppel Associated Box transcriptional repressor (dCas9-KRAB) and a genome-wide library of single guide RNAs (sgRNAs) to downregulate each of the ~20,000 human genes expressed in single-round HIV-GFP latently infected cell lines. Sorting the GFP+ cells allows PCR-amplification of the sgRNA sequences targeting potential HIV-1 latency promoting genes. These sequences are then inserted into an empty vector to generate an enriched sgRNA library. Serial application of REACT can unambiguously identify host genes that promote HIV-1 latency, even in the presence of high background “stochastic noise”. As a proof of concept, we applied REACT in the Jurkat-based 2D10 cell line, a widely used post-integration latency model where the d2EGFP reporter sequence is inserted in lieu of the viral *nef* gene in the proviral genome [36]. Both known and novel factors that promote HIV latency were identified using REACT in this cell line [33].

In the current study, we have advanced the use of REACT to identify human genes that enforce HIV latency at different integration sites in multiple cell lines, confirming results in a primary CD4 T cell model of HIV latency. Although favoring active genes [38], HIV integrates widely within the genome, often reflecting a varied chromatin landscape that influences its inducibility [39,40,41]. A key question is: Are there different sets of presently unrecognized host factors that operate in different integration sites and chromatin environments to determine the depth of latency? Insight into this question will be key for designing future therapeutic interventions that could involve sequential use of "shock and kill" and “block and lock” strategies.

## Results

### Construction of Doxycycline-Inducible CRISPRi Jurkat cell lines latently infected with HIV-GFP Exhibiting different levels of spontaneous and induced reactivation

**Fig 1:**
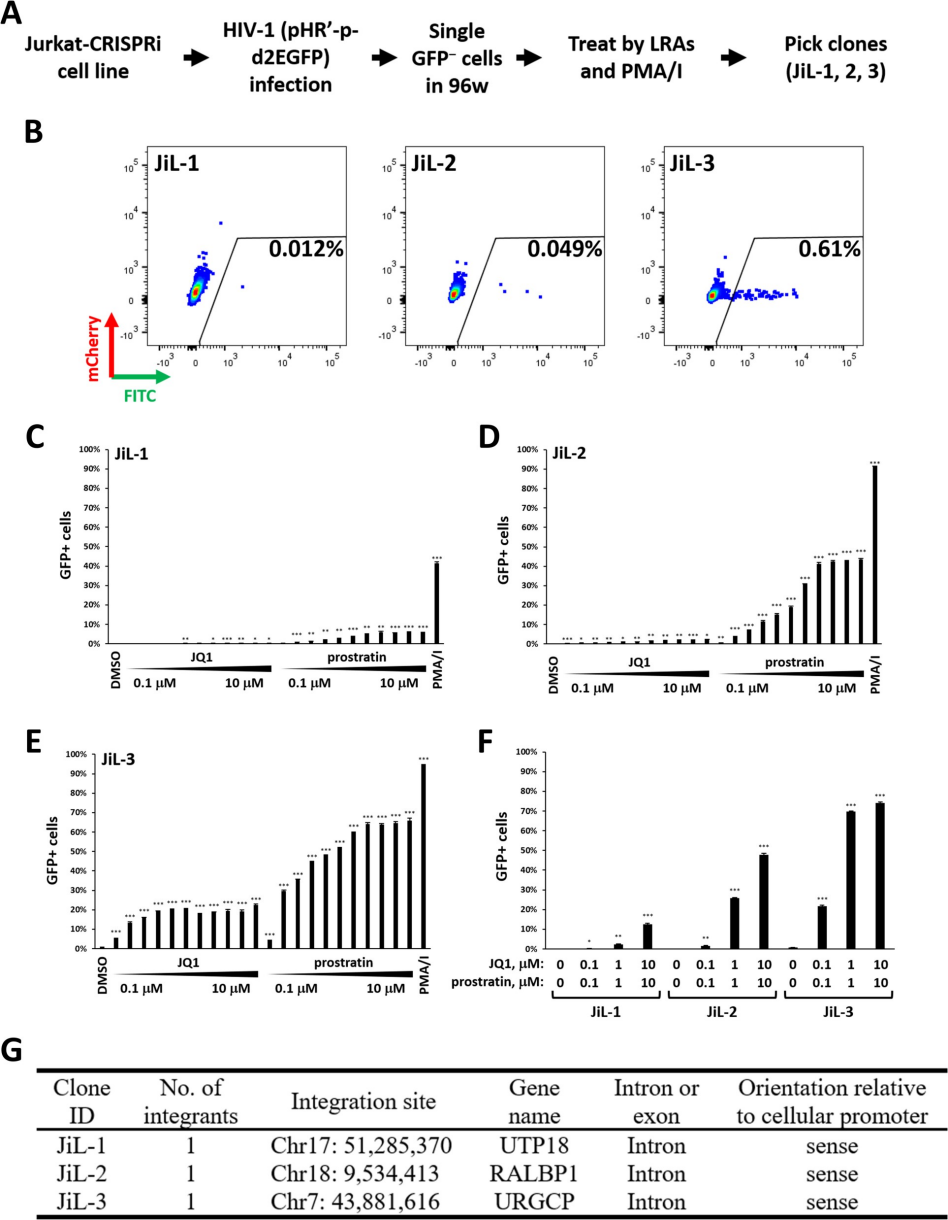
Construction of Doxycycline (Dox)-Inducible CRISPRi Jurkat Cell Lines Latently Infected with HIV-GFP Exhibiting Different Levels of Spontaneous and Induced Reactivation. **A.** Schematic summary of the procedure for constructing doxycycline-inducible CRISPRi Jurkat cell lines latently infected by HIV-GFP (“JiL” cell lines). **B.** Representative flow cytometry plots showing the spontaneous %GFP expression of the three JiL cell lines. **C., D., E., & F.** JiL-1, JiL-2 and JiL-3 cell lines were treated with: 0.1% DMSO, varying concentrations (0.1 μM, 0.2 μM, 0.4 μM, 0.6 μM, 0.8 μM, 1 μM, 2 μM, 4 μM, 6 μM, 8 μM, 10 μM) of JQ1 or prostratin, combinations of the two drugs, or 50 ng/ml (81 nM) PMA and 1 μM Ionomycin for 20 hours. The treated cells were then analyzed by flow cytometry to determine the percentages of GFP+ cells in each population. The GFP gate was designated based on uninfected Jurkat cells. Error bars represent mean +/− standard deviation (SD) from three experimental replicates. Asterisks denote levels of statistical significance compared with the DMSO groups calculated by two-tailed Student’s *t*-test (*: p<0.05, **: p<0.01, and ***: p<0.001). **G.** Proviral integration sites in JiL-1, JiL-2, and JiL-3 cells determined by inverse PCR.

To identify unrecognized host factors contributing to the maintenance of HIV latency at different integration sites, we first constructed a doxycycline (Dox)-inducible CRISPRi Jurkat cell line (named “Jurkat-CRISPRi”) where expression of the dCas9-KRAB fusion protein is induced by addition of Dox (S1 Fig). Using this cell line, we generated several J-Lat-like latently infected clones using a single-round HIV-d2EGFP reporter virus [36] based on previously described methods [37] (Fig 1A). Three of these cell lines (named "JiL-1", "JiL-2", and "JiL-3"; where “JiL” denotes “**J**urkat-**i**nducible CRISPRi-**L**atency”) exhibited different levels of spontaneous reactivation (Fig 1B) and responded differently when treated with two LRAs that activate HIV transcription via different mechanisms. These LRAs included JQ1 (BET inhibitor) and prostratin (protein kinase C activator), which were applied either alone or in combination (Fig 1C–1F). Notably, JiL-1 cells responded weakly to a combination of 1 μM JQ1 and 1 μM prostratin, and only moderately (12.6%) to 10 μM JQ1 and 10 μM prostratin that strongly activated (74%) of JiL-3 cells (Fig 1F). JiL-2 cells displayed a reactivation phenotype intermediate between JiL-1 and JiL-3.

A single HIV provirus was integrated at different chromosomal locations in each of the three cell lines (Fig 1G). We also treated two widely used latency model cell lines, J-Lat 6.3 [37] and 2D10 [36], with identical LRA concentrations and combinations. Results obtained with J-Lat 6.3 were similar to JiL-1, while results from 2D10 were similar to JiL-3 (S2 Fig). These results indicate that JiL-1, JiL-2, and JiL-3 represent different conditions where the inducibilities of the latent HIVs are respectively low, low-to-moderate, and high.

### Reiterative enrichment and authentication of CRISPRi targets (REACT) in JiL cell lines

**Fig 2:**
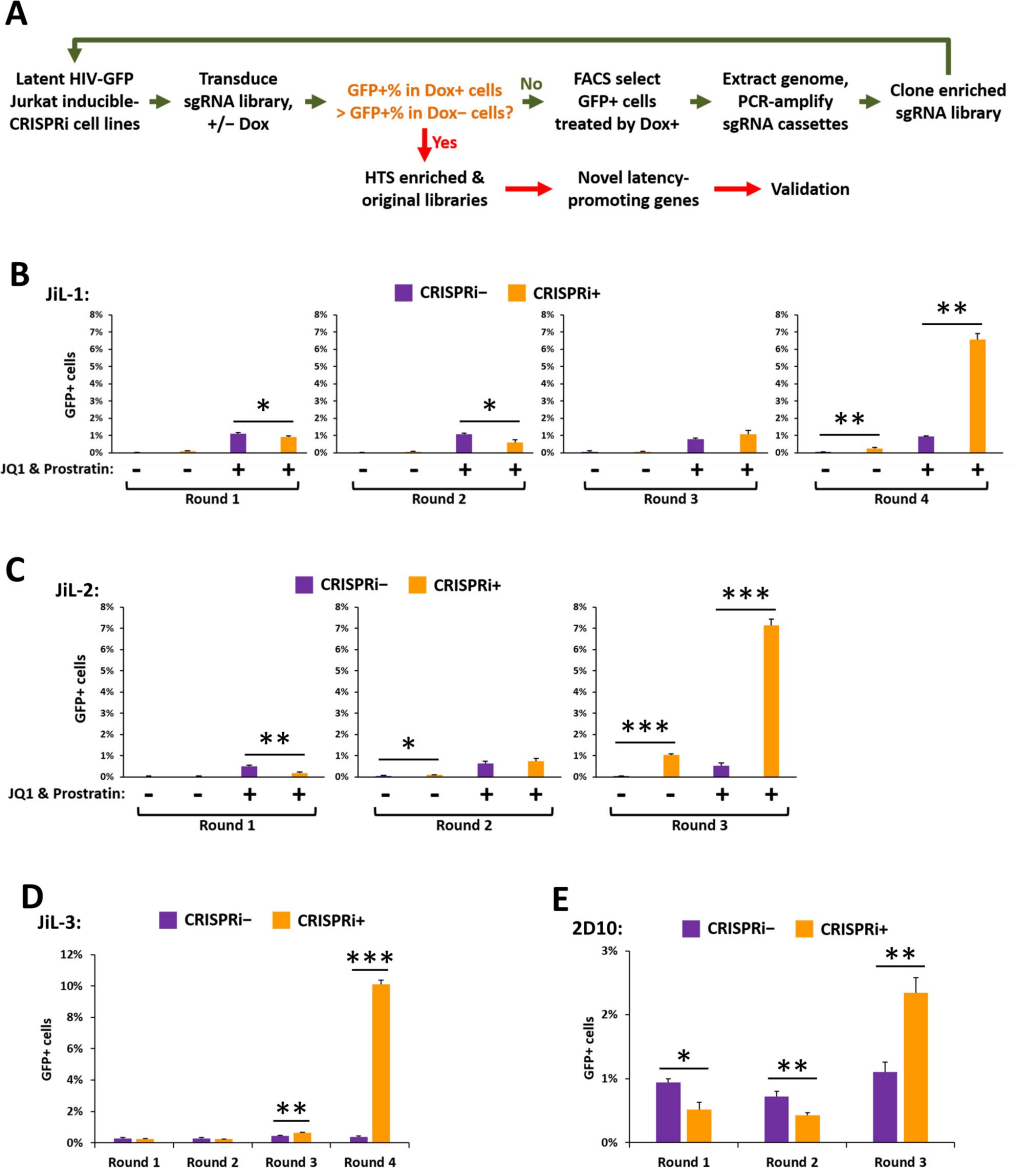
Reiterative Enrichment and Authentication of CRISPRi Targets (REACT) in JiL Cell Lines. **A.** Schematic summary of the progressive enrichment of the sgRNA sequences targeting latency-promoting genes and validation of the identified factors. **B., C., D., & E.** Flow cytometry results of the percentages of GFP+ cells in JiL-1, JiL-2, JiL-3 and 2D10-CRISPRi cells transduced by the original (in Round 1) or sequentially enriched sgRNA libraries. The cells were treated with either 0.1% DMSO (CRISPRi−) or 1 μg/ml Dox (CRISPRi+). Aliquots of JiL-1 and JiL-2 cells were also treated with 1 μM JQ1 + 0.2 μM prostratin or 0.1 μM JQ1 + 0.1 μM prostratin respectively to examine synergy between the enriched sgRNAs and the LRAs. Error bars represent mean +/− SD from three experimental replicates. Asterisks denote levels of statistical significance calculated by two-tailed Student’s *t*-test (*: p<0.05, **: p<0.01, and ***: p<0.001).

Next, the potential activity of unrecognized host factors enforcing latency in these cell lines was investigated using the REACT methodology [33] with minor modifications. Instead of the original sgRNA library [42] used in our previous study [33], a more compact and robust sgRNA library [43] was employed. This allowed scaling down the cell culture volume and simultaneous screening in multiple cell lines. As outlined in Fig 2A, four cell lines (JiL-1, JiL-2, JiL-3, and 2D10-CRISPRi [44]) were subjected to iterative CRISPRi screens. The sgRNA sequences were amplified from the genome of the FACS-sorted GFP+ cells, and cloned as an enriched sgRNA library, which was then transduced into the original cell line for the next round of selection. This process was reiterated until the enriched sgRNA library significantly increased the %GFP+ cells in the Dox treated cells (CRISPRi machinery expressed) compared to cells not treated with Dox. The sgRNAs enriched by the final round were then identified by high throughput sequencing, and the latency promoting function of the target genes were further validated in cell lines and primary cell models (Fig 2A). As demonstrated in Fig 2B–2E, after two to three rounds of screens, enriched sgRNA libraries notably improved the spontaneous activation and/or activation response to JQ1 and prostratin in each cell line. These results indicate that we have successfully enriched the sgRNAs that target key host latency promoting genes in each of the four cell lines.

**Fig 3:**
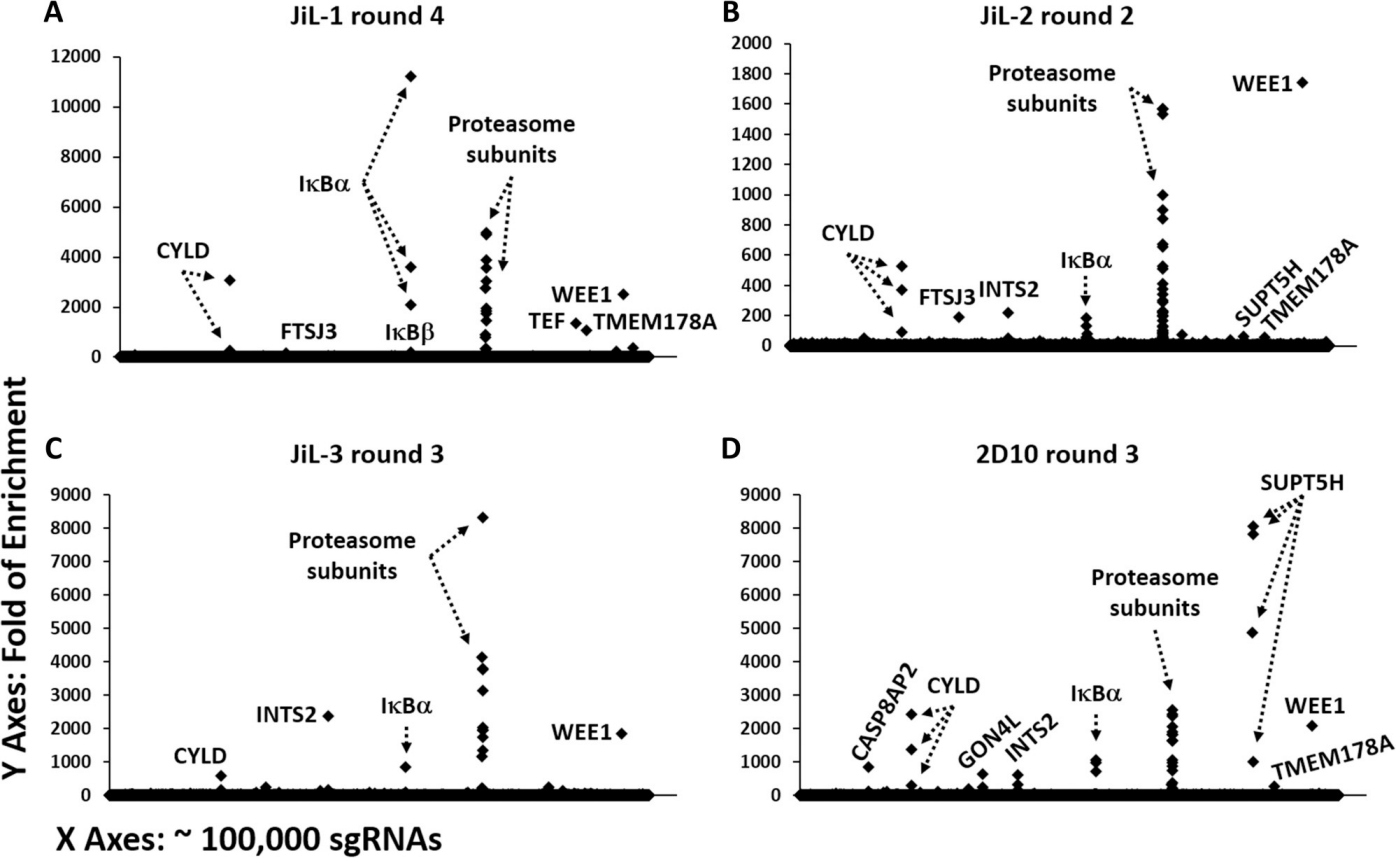
Overview of sgRNA Enrichment by REACT in Each Cell Line. **A., B., C., & D.** The original sgRNA library and the REACT-enriched sgRNA libraries were subjected to high throughput sequencing to calculate the folds of enrichment, which were then presented in scatter plots, with the genes targeted by the most-enriched sgRNAs labeled. CYLD, IκBα, and proteasomal subunits had been previously identified. Details regarding the folds of enrichment for the complete 104,534 sgRNAs in the four cell lines are provided in S1 Table.

Next, the sgRNA libraries were subjected to sequencing to identify which genes were targeted by the most enriched sgRNAs. A group of genes were repeatedly identified in all the four cell lines including: TMEM178A (a negative regulator of ER calcium fluxes that inhibits NFATc1 transcription factor activation [45]), FTSJ3 (an RNA 2′-O-methyltransferase interacting with TAR [46]), subunits of the Integrator complex INTS2, INTS5, INTS8 (multisubunit complex that interacts with HIV LTR and may cleave nascent mRNAs to induce premature transcription termination [47,48,49]) and NICN1 (a nuclear protein and part of the tubulin polyglutamylase complex that regulates intracellular trafficking and nuclear architecture [50,51,52,53]), as well as the previously reported IκBα, CYLD, and proteasome subunits [33,54,55,56], along with WEE1, a negative regulator of mitosis [57] (Fig 3, S1 Table). In contrast, the sgRNAs targeting SUPT5H, CASP8AP2, CHAF1A, and AZI2 were among the most-enriched in 2D10-CRISPRi cells, but only modestly enriched in JiL cell lines (Fig 3, S1 Table).

### Confirmation of host factors promoting HIV latency

**Fig 4:**
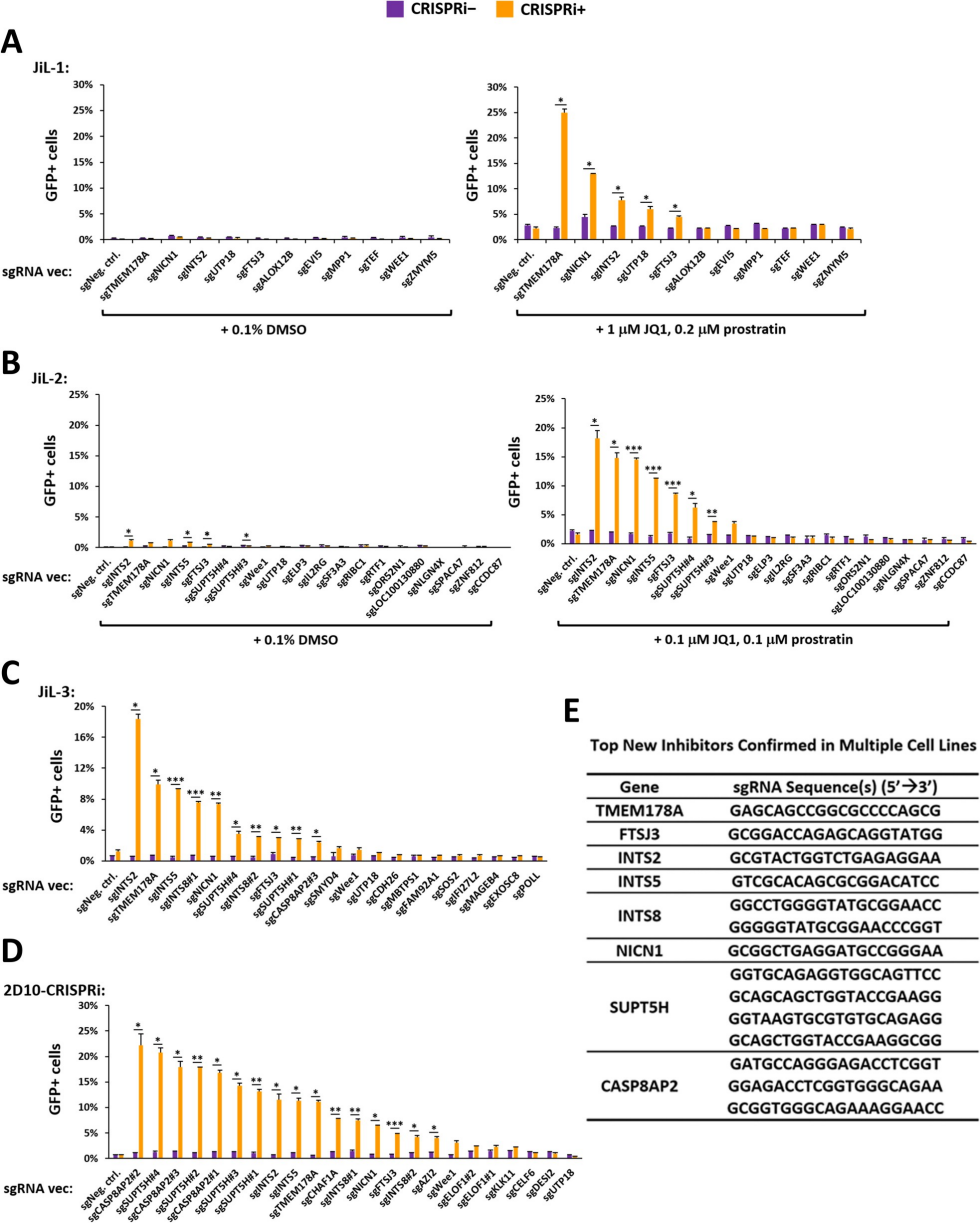
Individual Confirmation of Host Latency Promoting Factors that Decrease Inducibility of Latent HIV-1. **A., B., C., & D.** JiL-1, JiL-2, JiL-3 and 2D10-CRISPRi cells were transduced with the indicated sgRNA expression vectors, selected by puromycin, and then treated with either 0.1% DMSO (CRISPRi−) or 1 μg/ml Dox (CRISPRi+) for 3 days. Aliquots of JiL-1 and JiL-2 cells were also treated with the indicated concentrations of JQ1 and prostratin for 20 hours to examine synergy between the sgRNAs and the LRAs. The cells were then examined by flow cytometry to determine GFP+%. Error bars represent mean +/− SD from two experimental replicates. Asterisks denote levels of statistical significance calculated by two-tailed Student’s *t*-test (*: p<0.05, **: p<0.01, and ***: p<0.001). **E.** A list of the new HIV latency promoting gene products identified in this study and their corresponding sgRNAs confirmed to be effective in multiple cell lines.

The high throughput sequencing data allowed us to synthesize and clone the top sgRNA hits targeting genes that have not been reported as HIV latency promoting factors. To validate their activity, individual sgRNAs were introduced into the cell lines with and without Dox treatment and the spontaneous levels of reactivation alone or in the presence of JQ1 and prostratin were assessed (Fig 4). The sgRNAs targeting TMEM178A, FTSJ3, subunits of the Integrator complex (INTS2, INTS5, INTS8), and NICN1 enhanced the response of JiL-1 and JiL-2 to combinations of prostratin and JQ1 (Fig 4A and 4B) or spontaneous GFP expression in JiL-3 cells (Fig 4C). RT-qPCR assays demonstrated that these sgRNAs significantly downregulated expression of the respective target gene RNA and increased levels of HIV-1 *env* mRNA (S3 Fig).

We further confirmed the repressive activity of these four gene products using shRNAs for knockdown (S4A Fig) with a concomitant increase in activity of the HIV-GFP reporter virus (S4B Fig). In contrast to the JiL cell lines, 2D10-CRISPRi cells were most induced by sgRNAs targeting SUPT5H and CASP8AP2 (Fig 4D). About one third to half of the tested sgRNAs failed to increase HIV inducibility despite high level enrichment in the cell lines. Of note, an sgRNA targeting UTP18 proved effective only in JiL-1 cells with no effects in JiL-2, JiL-3, or 2D10-CRISPRi cells (Fig 4A and 4D). An shRNA targeting UTP18 only weakly increased HIV inducibility in JiL-1 cells (S4 Fig). Of note, in JiL-1 cells, the HIV provirus is integrated within the UTP18 gene suggesting that transcriptional interference may play a role in the observed latency. Finally, the sgRNA targeting WEE1 failed to increase HIV inducibility in any of the cell lines. In summary, based on the results of iterative CRISPRi screens, we have identified and confirmed eight genes that promote HIV latency in multiple Jurkat-based cell lines (Fig 4E).

### The host latency promoting factors, FTSJ3, INTS2, NICN1, and TMEM178A, also contribute to HIV latency in primary CD4 T cells

**Fig 5:**
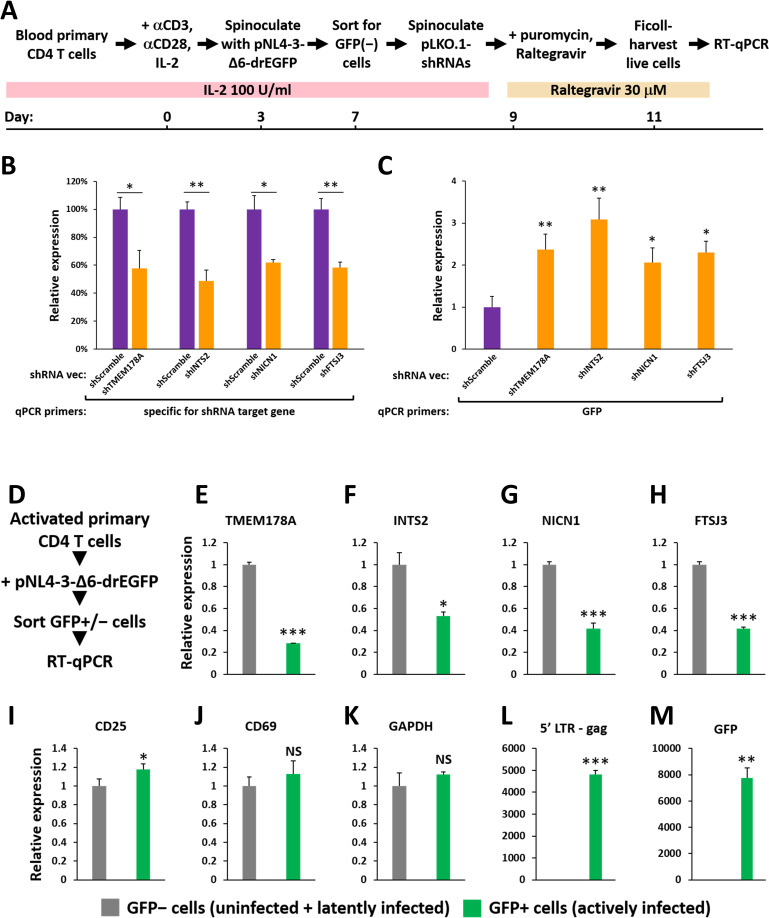
Host Latency Promoting Factors, FTSJ3, INTS2, NICN1, and TMEM178A, Contribute to HIV Latency in Primary Human CD4 T Cells. **A. & D.** Schematic summaries of the protocols used for testing the function of host factors in maintaining HIV latency (A) and correlation of the expression levels of these host factors with the ability of primary CD4 T cells to support active HIV infection (D). **B., C., E., F., G., H., I., J., K., L., & M.** RT-qPCR analyses of the mRNA levels of the genes that are denoted by their corresponding qPCR primers or are labeled on top of the panel. In panels B–C, the mRNA level detected in the shScramble-transduced cells was set to 1; in panels E–M, mRNA level detected in the GFP− cells was set to 1. Error bars represent mean +/− SD from three experimental replicates. Asterisks denote levels of statistical significance calculated by two-tailed Student’s *t*-test (*: p<0.05, **: p<0.01, and ***: p<0.001).

Among the eight factors identified (Fig 4E), FTSJ3, NICN1, TMEM178A, and subunits of the Integrator complex have not been reported as HIV latency promoting factors. To validate their function, we first assessed their level of expression in primary blood CD4 T cells. As shown in S5 Fig, FTSJ3, NICN1, TMEM178A, and INTS2 are highly expressed in unstimulated primary CD4 T cells. However, after T cell activation, levels of expression for each declined, but were still comparable to or higher than that present in the Jurkat cells. Based on the latency-promoting function of these genes in the JiL cell lines, we asked whether the high-level expression of these genes could enforce HIV latency in primary CD4 T cells. Specifically, we tested whether downregulation of these four genes resulted in enhanced HIV replication in primary CD4 T cells. Activated primary CD4 T cells were infected with single-round CXCR4-tropic HIV-1 pseudoviruses [58], GFP− cells were sort-purified 4 days post infection, then each of the four genes were knocked down using pLKO.1-shRNA lentiviral vectors. shRNA+ cells were selected by addition of puromycin, and then Ficoll-harvested live cells were subjected to RT-qPCR (Fig 5A). Our results indicated that the shRNAs induced a 40–50% decrease in expression of FTSJ3, NICN1, TMEM178A, and INTS2 (Fig 5B), and significantly enhanced HIV expression in primary CD4 T cells by 2- to 3- fold compared to effects of the shScramble control shRNA (Fig 5C). These findings support the function of FTSJ3, INTS2, NICN1, and TMEM178A as HIV latency promoting factors in primary CD4 T cells.

Next, expression levels of these four host latency promoting factors were determined to investigate whether they correlated with the ability of primary CD4 T cells to support active HIV infection. We first infected the activated primary CD4 T cells with single-round CXCR4-tropic HIV-1 pseudoviruses [58], sorted GFP+ and GFP− cells 4 days post infection, and then conducted RT-qPCR in these two groups of cells (Fig 5D). The results indicate that in GFP+ cells (actively infected as demonstrated by RT-qPCRs targeting an LTR-gag region and GFP in Fig 5L–5M), the expression levels of FTSJ3, NICN1, TMEM178A, and INTS2 were significantly lower than in the GFP− cells (uninfected/latently infected) (Fig 5E–5H). Of note, the expression levels of the T cell activation markers CD25, CD69 and the housekeeping gene, GAPDH, in the GFP+ cells were similar as those in the GFP− cells (Fig 5I–5K). These results indicate that, in activated CD4 T cells, active HIV infection may preferentially occur in a subpopulation of cells expressing lower levels of these four latency promoting factors.

### Downregulation of FTSJ3, INTS2, NICN1, and TMEM178A stimulate different stages of RNA polymerase II-mediated transcription of HIV-1

**Fig 6:**
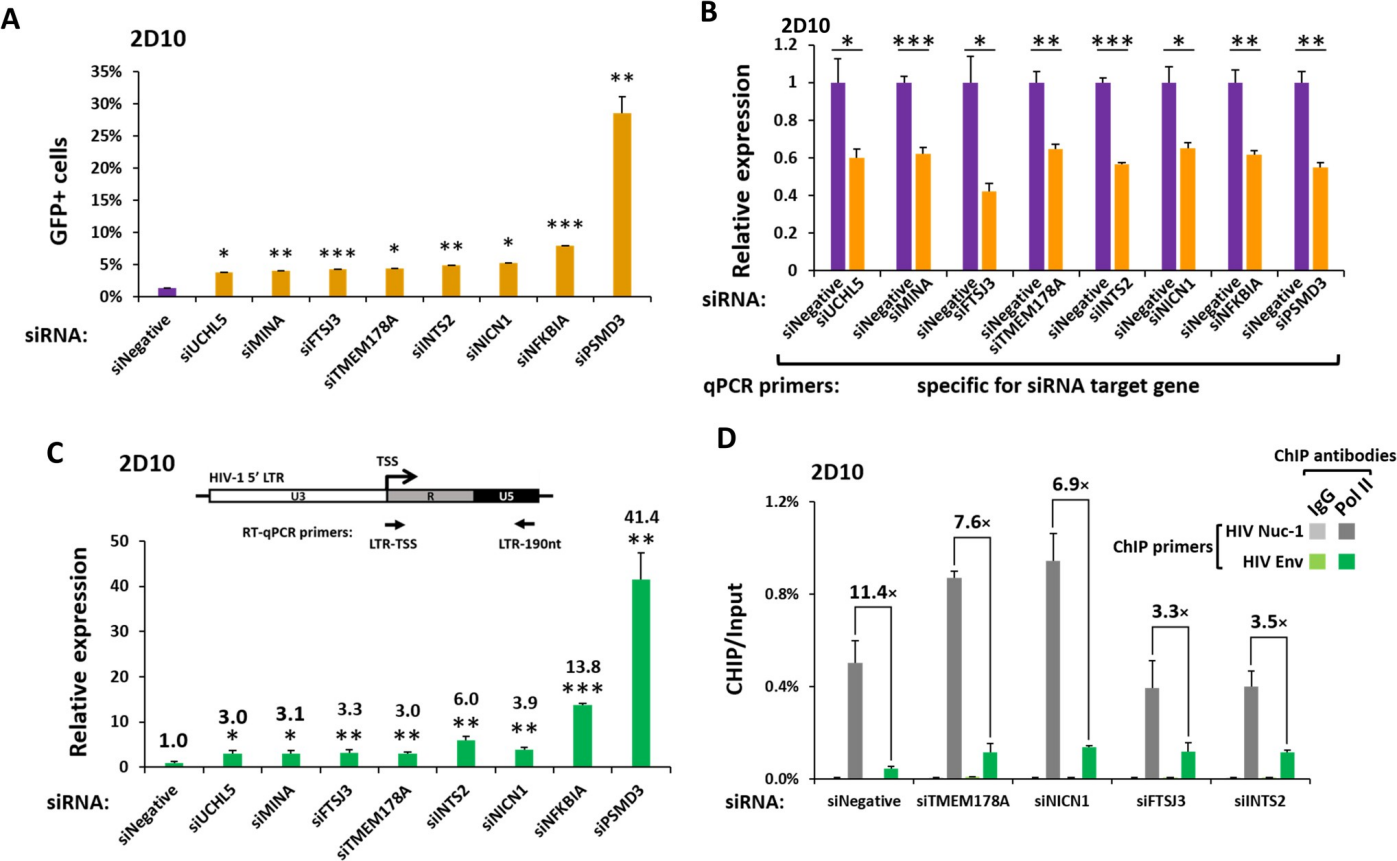
Downregulation of FTSJ3, INTS2, NICN1, and TMEM178A Stimulate Different Stages of RNA Polymerase II Transcription of HIV-1. Jurkat 2D10 cells were nucleofected with either non-targeting siRNA (siNegative) or siRNAs targeting the indicated genes and subjected to: **A.** Flow cytometry to determine the percentages of GFP+ cells in each population. **B. & C.** RT-qPCR analyses of the mRNA levels of the genes or region within the HIV provirus that are denoted by their corresponding qPCR primers. All qPCR signals were normalized to those of beta-actin. In panel B, the mRNA levels detected in the CRISPRi− cells were set to 1; in panel C, the mRNA level detected in the siNegative cells was set to 1, and the relative expression level in each group was labeled. **D.** ChIP-qPCR analyses to determine the levels of RNA polymerase II bound to the proximal HIV LTR or more distant Env region. The ChIP-qPCR signals were normalized to those of input DNA. In all panels, error bars represent mean +/− SD from three experimental replicates. In panels A–C, the asterisks indicate the levels of statistical significance calculated by two-tailed Student’s *t*-test (*: p<0.05, **: p<0.01, and ***: p<0.001); in panel D, the ratios between the indicated signals are indicated.

The productive transcription of HIV requires concerted actions of host factors to enable both initiation and elongation stages by RNA polymerase II (Pol II) on the HIV LTR [6,59]. To better understand how the newly identified host inhibitors are acting, siRNAs were used to knock down the expression of each in Jurkat 2D10 cells (Fig 6B). Consistent with the results in JiL cells obtained with sgRNAs (Fig 4) and shRNAs (S4 Fig), siRNAs targeting FTSJ3, INTS2, NICN1, and TMEM178A significantly reactivated latent HIV in 2D10 cells (Fig 6A). siRNA knockdown of four previously reported inhibitors of HIV, UCHL5 (UCH37) [56], MINA (MINA53) [60], NFKBIA and PSMD3 [33] produced comparable levels of HIV reactivation except PSMD3 knockdown appeared more potent (Fig 6A). Using an RT-qPCR assay with a pair of primers that anneal to transcription start site (TSS) and a region downstream of the Pol II pausing site, we found that downregulation of these host inhibitors significantly increased the elongated HIV mRNA (Fig 6C), indicating enhancement of productive Pol II transcription from the HIV LTR.

To examine the different stages of Pol II transcription on HIV LTR regulated by the four new host inhibitors, we conducted chromatin immunoprecipitation (ChIP) assays using the siRNA-nucleofected 2D10 cells. Downregulation of TMEM178A and NICN1 increased the Pol II signals both on HIV LTR (Nuc-1) and within Env region of the provirus suggesting effects at both the levels of Pol II initiation and elongation. Downregulation of INTS2 and FTSJ3 increased Pol II detection in the Env gene but not in the proximal LTR (Fig 6D) indicating a primary effect at the level of Pol II elongation. Consistent with major effects at the level of elongation, siFTSJ3 and siINTS2 decreased the pausing index more than siNICN1 and siTMEM178A did (Fig 6D, numbers on top of the bars). These results indicate that elongation is likely to be the main step in HIV transcription regulated by INTS2 and FTSJ3, while initiation is likely to be the principal step regulated by TMEM178A and NICN1.

## Discussion

**Fig 7:**
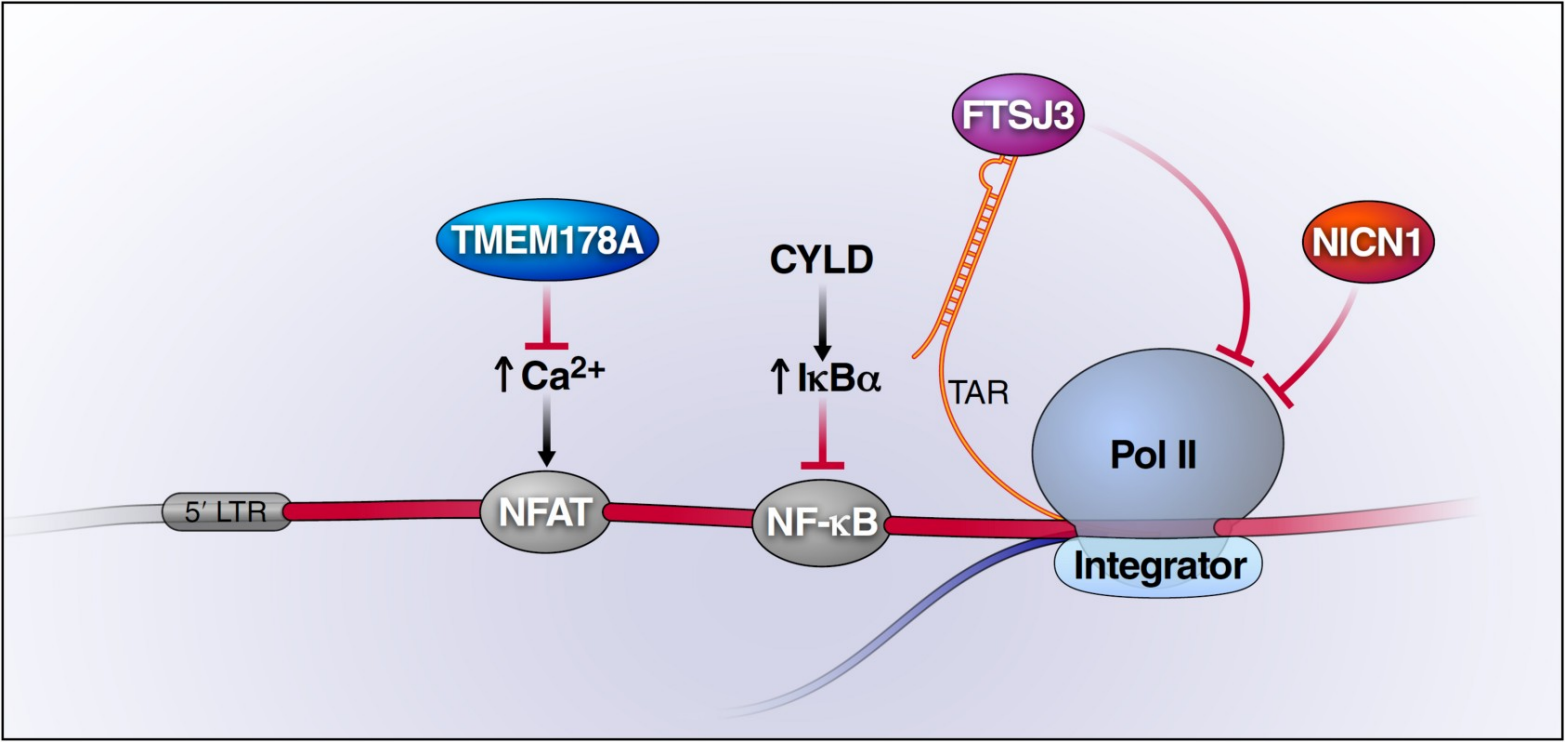
Graphic Summary of the Likely Action of New Host Latency Promoting Factors Identified in This Study. The potential mechanisms depicted are based on the previously reported mechanisms of action of these factors. See Discussion for more details.

In summary, using an iterative CRISPRi screening strategy termed REACT [33], we identify four previously unrecognized host factors that enhance HIV-1 latency. Our current findings suggest that these factors likely block HIV transcription through different mechanisms (Fig 7). TMEM178A negatively regulates ER calcium fluxes thus inhibiting NFATc1 [45], one of the key factors that initiates HIV proviral transcription [61]. The Integrator complex associates with HIV LTR [47] and its ability to cleave nascent mRNAs to induce premature transcription termination of promoter-proximally paused RNA polymerase II in *Drosophila* cells [48,49], if conserved in human cells, may lead to abortive HIV transcription initiation. FTSJ3 methylates nascent HIV genome by interacting with TAR [46], where it may interfere with the functioning of transcription machinery. NICN1 is a nuclear protein and part of the tubulin polyglutamylase complex [50,51] that regulates intracellular trafficking and nuclear architecture [52,53]. Of note, these four factors enforce HIV latency in all four cell lines used in our REACT screens, indicating their fundamental importance regardless of the integration site. Supporting this notion, individual downregulation of these four factors significantly enhanced HIV expression in primary CD4 T cells with different integration sites (Fig 5).

Alongside these four factors, our screens also recognized factors that regulate HIV latency in a cell line/integration site-specific manner. The 2D10 cell line was developed by progressive shutdown of active HIV transcription, a long (> 3 weeks) process during which blockade of transcription elongation and the resulting decline of the Tat-feedback circuit plays a critical role in establishing latency [36]. Consistent with this mechanism, sgRNAs targeting the negative elongation factors SUPT5H and NELFA were highly enriched in 2D10 cells (S1 Table). In contrast, in the JiL cell lines, which were generated by isolating clones from the population of cells that were transcriptionally silenced shortly (4 days) after infection (Fig 1A and Materials and Methods), the sgRNAs targeting SUPT5H and NELFA were enriched to a much lesser extent (S1 Table). These findings suggest a smaller contribution of transcriptional pausing to HIV latency occurring in these cell lines. In addition, we find that sgRNAs targeting CASP8AP2, which has previously been reported as an inhibitor of acute HIV infection in HEK293T cells [54], strongly reactivates latent HIV in 2D10 cells and to a lesser extent in the JiL-3 cells (Fig 4C and 4D). Of note, Hsiao et al. recently reported that IL-7 receptor-alpha (CD127) memory CD4+ T cells from tonsil tissue preferentially support latent HIV-1 infection [62]. In their RNA-seq data, two host repressors identified in this study, INTS8 and CASP8AP2 were preferentially expressed in the CD127+ tissue memory CD4+ T cells relative to the CD57+ cells that support active HIV replication. These results suggest that the mechanisms that maintain HIV-1 latency may be partly conserved between CD4+ T cells in blood and lymphoid tissues, and that the increased expression of these two proteins may contribute to the propensity of CD127+ cells to undergo latent HIV-1 infection.

It is important to note that the reporter viruses [36,58] we used for screening in Jurkat cells and the validation studies in primary cells are *nef*-deficient. Given its essential roles in the full life cycle and pathogenesis of HIV [63,64,65,66,67], it remains to be seen whether Nef antagonizes the function of one or more of these latency-promoting genes, and whether these latency-promoting genes also block reactivation of wild-type HIV.

We also identified a strictly integration site-specific latency-promoting gene, UTP18, whose sgRNA is effective in JiL-1, but not the other JiL lines and 2D10 cells (Fig 4A–4D). Since HIV is integrated between exons 10 and 11 of the UTP18 gene in JiL-1 cells (Fig 1G), the integration site-specific enforcement of HIV latency by UTP18 in this cell line seems likely due to transcriptional interference [7]. Consistent with this possibility, shRNAs targeting UTP18 fail to significantly increase HIV inducibility in JiL-1 cells (S4 Fig) because they act too late.

Methodologically, REACT provides a potent means to enrich genotypes underlying stochastic phenotypes, like reactivation of latent HIV. These phenotypes are inherently noisy because the genes underlying such phenotypes regulate their probability, rather than determining their occurrence [34]. Compared with traditional pooled genetic screens [42,68,69], the progressive enrichment process of REACT allows the study of stochastic phenotypes despite their inherently high background noise.

Recently, multiple laboratories have conducted genetic screens based on CRISPR-knockout (KO) or RNAi techniques to identify host genes involved in the regulation of HIV latency. Comparing the results from three prior studies [56,60,69] with our study (S1 Table), we find that 14 genes identified as top latency-promoting genes in the previous screens rank within top 5% in at least one of the four cell lines employed in our screens: ELP3, CNOT6, POLE3, KDM1A, MFN2, PROCR (identified by [56]); FOXP1, KAT7, UBA5, PRKRIR, TOPBP1, NFE2L3 (identified by [60]); KRTAP5-1, ABI1 (identified by [69]) (S2 Table). Reminiscent of the lack of overlap among the top hits in these previous studies, none of the top new host inhibitors we now identify (Fig 4E) ranks within top 10% in the published data sets from the prior studies (S3 Table). This kind of variation could result from differences in screening techniques. CRISPR-KO using wildtype Cas9 [56,60] generates complete knockouts that may lead to more robust phenotypes. In comparison, CRISPRi and RNAi [69] generate partial knockdowns that may permit identification of essential genes lost with complete knockouts. Compared with previous RNAi screens [69], the iterative CRISPRi strategy used in this study strongly enriches authentic hits (Fig 3) but may under-sample sgRNAs that inhibit cell proliferation. Therefore, REACT, single-round CRISPRi/RNAi, and CRISPR-KO screens are complementary and, when consolidating the results, may generate a more complete picture of the mechanisms regulating HIV latency.

## Materials and methods

### Generation of Jurkat inducible CRISPRi HIV-1 Latency (JiL) cell lines

All Jurkat-based cells were maintained in RPMI 1640 medium with L-glutamine (Gibco 11875), 10% fetal bovine serum (Gemini 100–106), 100 IU/ml penicillin and 100 μg/ml streptomycin (Gibco 15140) in 5% CO_2_, 90% relative humidity at 37°C. To set up the inducible CRISPR interference (CRISPRi) platform, Jurkat cells (from American Type Culture Collection) were transduced by the lentiviral vectors pHR-TRE3G-Krab-dCas9-P2A-mCherry [42] and pLVX-Tet-On Advanced [70]. A clone (named “Jurkat-CRISPRi”) expressing KRAB-dCas9-HA and mCherry in response to doxycycline (Dox) was first selected by fluorescence-activated cell sorting (FACS) and then verified by Western blotting.

Establishment of latent HIV-1 infection in Jurkat-CRISPRi cells was based on previous protocol [37] with modifications. Briefly, VSV-G pseudotyped single-round HIV-1 viral particles were packaged in HEK 293T cells (from American Type Culture Collection) using a 3rd generation lentiviral packaging system [71] with the pHR′-p-d2EGFP construct containing wild-type *tat*, *rev*, *env*, *vpu*, and the reporter gene d2EGFP in place of *nef* [36]. The Jurkat-CRISPRi cells were infected by the viral particles at an effective multiplicity of infection of 0.1 and kept in culture for four days. The GFP-negative (GFP−) cells were then sorted by FACS into 96-well plates at one cell per well to generate clonal cell lines. Two weeks after the sorting, the percentage of GFP-positive (GFP+) cells within these clones were examined by FACS after treatment by DMSO, PMA + Ionomycin, JQ1, or prostratin. Three clones (JiL-1, JiL-2, and JiL-3) with different profiles of response were selected, and their proviral integration sites determined by inverse PCR [36,72].

### Reiterative enrichment and authentication of CRISPRi targets (REACT) in JiL cell lines

JiL-1, JiL-2, JiL-3 (generated in this study), and 2D10-CRISPRi (previously generated by Dr. Qiang Zhou’s laboratory [44]) cell lines were in parallel subjected to REACT based on our recently developed strategy [33] with modifications. Instead of the original sgRNA library [42] employed in our previous study [33], a more compact and robust sgRNA library (CRISPRi-v2, containing ~100,000 sgRNA sequences, 5 sgRNAs/gene) [43] was used. The CRISPRi-v2 sgRNA library, which is in a pSico-based vector with BFP marker and puromycin-resistance (pSico-BFP-puro), were packaged in HEK 293T cells and transduced into 1×10^8^ of each of the four CRISPRi cell lines at an effective multiplicity of infection of less than one sgRNA per cell. Two days after transduction, the non-transduced cells were killed by adding puromycin into the medium at a final concentration of 1 μg/ml. The viability, BFP+%, and cell number were then checked daily by FACS, and the total number of BFP+ cells were constantly kept at 1×10^8^–1.5×10^8^ to maintain a library coverage of at least 1,000 cells per sgRNA. When the cells’ viability reached 70% and BFP+% reached 95%, about 5×10^7^ of the live cells were treated with 1 μg/ml Dox for three days in 500 ml medium. On the second day of Dox treatment, 1 μM JQ1 + 0.2 μM prostratin and 0.1 μM JQ1 + 0.1 μM prostratin were added to the medium of JiL-1 and JiL-2 cells, respectively. On day three, the cells (about 3×10^8^ at this time) were selected by FACS for the GFP/mCherry/BFP triple-positive phenotype.

The sgRNA cassettes were then PCR-amplified using the primer pair REACT-5F/REACT-3R [33] from the genomes of the triple-positive cells, digested with BstXI (Thermo Scientific FD1024) and Bpu1102I (Thermo Scientific FD0094), cloned into the empty library vector pSico-BFP-puro, amplified in *E*. *coli*, extracted as an enriched library and then re-transduced into the original JiL-1, JiL-2, JiL-3, or 2D10-CRISPRi cells for the next round of REACT. During each round of REACT, an aliquot of puro-selected cells was treated with DMSO instead of Dox and/or LRAs to serve as negative controls for FACS gating and monitoring in real-time the effectiveness of each round of REACT, which was reiterated for each cell line until the enriched library significantly enhanced HIV re-activation. At this point, the sgRNA sequences from the enriched libraries and the original library were determined by high throughput sequencing and analyzed as previously described [33]. The most-enriched sgRNAs were individually cloned into pSico-BFP-puro, and their latency-reversing effects independently assessed.

### Cell line-based latency reversal assay

The Jurkat-based latency models, including JiL cells (generated in this study), 2D10 (previously generated by Dr. Jonathan Karn’s laboratory [36]), and J-Lat 6.3 (previously generated by Dr. Eric Verdi’s laboratory [37]) were treated with various LRAs at the indicated concentrations for 20 hours. To induce CRISPRi, the cells were treated by 1 μg/ml Dox for 3 days. For control groups, 0.1% DMSO was used. Cells were then re-suspended in cold phosphate-buffered saline (PBS). Quantification of the GFP+ cells was performed using a BD Bioscience LSR Fortessa X20 cytometer. The data were analyzed with the Flowjo software and plotted as bar graphs with error bars representing standard deviations.

### Latency reversal assay in latently infected primary CD4 T cells

Concentrated blood from healthy volunteers were obtained in leukoreduction system chambers from Vitalant (https://www.vitalant.org), and peripheral blood mononuclear cells (PBMCs) were isolated using Lymphoprep medium (STEMCELL 07851). CD4 T cells were isolated from PBMCs using negative selection by EasySep kit (STEMCELL 19052) according to the manufacturer’s instructions and cultured at 10^6^ cells/ml in STCM medium [73] containing 100 U/ml IL-2 (R&D Systems 202-IL), or frozen at -80°C and then thawed in STCM one day before the subsequent experiments.

On day 0, the primary CD4 T cells were activated by 1 μg/ml anti-CD28 antibody (BD Biosciences 555725) in 6-well plates coated with anti-CD3 antibody (BD Biosciences 555336). The recombinant CXCR4-tropic HIV-1 pseudoviruses were generated by co-transfecting HEK293T cells with a plasmid encoding the HIV-1 envelope (pCXCR4), pC-Help, and pNL4-3-Δ6-drEGFP [58]. pLKO.1-puro lentiviral vectors containing the shRNA cassettes (S4 Table) were also packaged in HEK293T cells using a 3rd generation lentiviral packaging system [71]. On day 3, supernatant of HIV-1 was harvested, spun at 350 g, and spinoculated into the activated CD4 T cells by centrifugation at 800 g for 2 hours at 30°C with 6 μg/ml polybrene (Santa Cruz sc-134220). The spinoculated cells were then resuspended at 10^6^ cells/ml in STCM containing 100 U/ml IL-2. On day 7 (4 days after HIV infection), the GFP+ and GFP− cells were sorted on a BD Aria II flow cytometer using uninfected cells as control. The GFP+ cells and an equal number of the GFP− cells were subjected to RT-qPCR. The remaining GFP− cells were subjected to spinoculation of the shRNA lentiviral supernatants. On day 9 (2 days after shRNA transduction), puromycin (Sigma P8833) and raltegravir (Sigma CDS023737) were added to the medium of the shRNA-transduced cells to a final concentration of 5 μg/ml and 30 μM, respectively. On day 11 (2 days after adding puromycin), live cells were harvested by Ficoll centrifugation and subjected to RT-qPCR to quantify knockdown efficiency and HIV transcription.

### Reverse transcription and quantitative PCR (RT-qPCR) assay

Total cellular RNAs were extracted by using TRIzol Reagent (Invitrogen 15596) and reverse transcribed by M-MLV Reverse Transcriptase (Promega M1701) with random hexamers (Invitrogen 48190). The cDNAs were then subjected to qPCR using DyNAmo HS SYBR Green qPCR kit (Thermo Scientific F-410L) on a CFX384 system (Bio-Rad) with the primer pairs listed in S4 Table. All reactions were carried out in triplicates. The PCR signals were normalized to those of beta-actin and displayed as bar graphs with error bars representing standard deviations.

### siRNA nucleofection in Jurkat 2D10 cells

The following siRNAs were purchased from Thermo Fisher: siNegative (4390843), siNFKBIA (s9512), siPSMD3 (s11393), siMINA (s39524), siUCHL5 (s28048), siINTS2 (s33184), siTMEM178A (s43535), siNICN1 (s38792), siFTSJ3 (s42146). For every 10^6^ Jurkat 2D10 cells 30 pmol siRNA was introduced using SE Cell Line 4D-Nucleofector X Kit L (Lonza V4XC-1024) according to the manufacturer's protocol (program CL-120) on a 4D-Nucleofector X Unit (Lonza AAF-1002X). Two days after nucleofection, 10^6^ cells were subjected to flow cytometry and RT-qPCR assays as described above, and 3 × 10^6^ cells were subjected to ChIP assays as described below.

### Chromatin immunoprecipitation (ChIP) assay

Jurkat 2D10 cells were fixed with 1% formaldehyde for 10 min at room temperature, and ChIP assays were performed using the ChIP-IT Express Enzymatic kit (Active Motif 53009) according to the manufacturer's protocol. For each ChIP reaction, 2 μg of either normal rabbit IgG (Bethyl P120-101) or rabbit anti-human RNA Polymerase II antibody (Bethyl A304-405A) were added. qPCR was performed with the primers listed in S4 Table. Signals obtained by qPCR were normalized to those of input DNA, and the averages from triplicate qPCR reactions were used to generate standard deviation depicted by the error bars. Two-tailed Student's *t*-tests were conducted, and the different significance levels were marked by 1 to 3 asterisks (*: p<0.05, **: p<0.01, and ***: p<0.001).

## Supporting information

S1 Fig Characterization of the Doxycycline (Dox)-inducible CRISPRi Jurkat Cell Line (named “Jurkat-CRISPRi”) Constructed in This Study. **A.** Results of Western blot analyses of the Dox-inducible expression of the KRAB-dCas9-HA fusion protein in the whole cell lysates (WCL) of Jurkat-CRISPRi cells. **B.** Representative FACS plots showing the Dox-inducible expression of the mCherry fluorescent protein as a reporter for the expression of KRAB-dCas9-HA fusion protein in the Jurkat-CRISPRi cells. In both panels, the cells were treated with either 0.1% DMSO (CRISPRi−) or 1 μg/ml Dox (CRISPRi+) for 2 days.
(PDF)

S2 Fig Characterization of the Response of J-Lat 6.3 and 2D10 Cell Lines to LRAs. **A., B., & C.** The J-Lat 6.3 and 2D10 cell lines were treated with 0.1% DMSO, varying concentrations (0.1 μM, 0.2 μM, 0.4 μM, 0.6 μM, 0.8 μM, 1 μM, 2 μM, 4 μM, 6 μM, 8 μM, 10 μM) of JQ1 or prostratin, combinations of the two drugs, or 50 ng/ml (81 nM) PMA and 1 μM Ionomycin for 20 hours. The treated cells were then subjected to FACS analyses to determine the percentages of GFP+ cells in each population. Error bars represent mean +/− standard deviation (SD) from three experimental replicates.
(PDF)

S3 Fig Confirmation of the sgRNAs’ Ability to Downregulate Target Genes and Increase HIV mRNA. **A., B., C. & D.** RT-qPCR analyses of the mRNA levels of the genes that are denoted by the corresponding qPCR primers. The JiL cells were first transduced with the indicated sgRNA vectors, selected in the presence of puromycin, and then treated with either 0.1% DMSO (CRISPRi−) or 1 μg/ml Dox (CRISPRi+) for 3 days. The JiL-1 cells were also treated by 1 μM JQ1 + 0.2 μM prostratin for 20 hours before analyses. The mRNA levels detected in the CRISPRi− cells were set to 1. Error bars represent mean +/− SD from three experimental replicates. Asterisks denote levels of statistical significance calculated by two-tailed Student’s *t*-test (*: p<0.05, **: p<0.01, and ***: p<0.001).
(PDF)

S4 Fig shRNAs Targeting TMEM178A, NICN1, INTS2, and FTSJ3 Enhance Reactivation of Latent HIV in JiL-1 Cells. The JiL-1 cells were first transduced with the indicated shRNA vectors, and then selected by 1 μg/ml puromycin for 3 days. **A.** The cells were then subjected to RT-qPCR analyses of the mRNA levels of the genes that are denoted by their corresponding qPCR primers. **B.** All the cells were treated by 1 μM JQ1 + 0.2 μM prostratin for 20 hours, and then examined by flow cytometry for GFP+%. In both panels, error bars represent mean +/− SD from three experimental replicates. Asterisks denote levels of statistical significance calculated by two-tailed Student’s *t*-test (*: p<0.05, **: p<0.01, and ***: p<0.001).
(PDF)

S5 Fig Relative Expression Levels of FTSJ3, NICN1, TMEM178A, and INTS2 in Resting and Activated Primary CD4 T Cells Compared to Jurkat Cells. RT-qPCR analyses of the mRNA levels of the indicated genes in Jurkat cells and primary CD4 T cells before and after activation by antibodies against CD3 and CD28. To allow comparison, the mRNA level detected in the Jurkat cells was set to 1. Error bars represent mean +/− SD from three experimental replicates.
(PDF)

S1 Table Fold of Enrichment for Each sgRNA by REACT in JiL-1, JiL-2, JiL-3, and 2D10-CRISPRi Cells. (XLSX)

S2 Table How the Top Latency-promoting Genes Identified in Three Previous Studies Rank in This Study. (XLSX)

S3 Table How the Top New Host Inhibitors Identified and Confirmed in This Study Rank in the Published Data Sets from Three Previous Studies. (XLSX)

S4 Table List of DNA Oligonucleotides Used in This Study. (DOCX)
