# Supplementary figures and images for: Identification of unrecognized host factors promoting HIV-1 latency
Source: PLoS Pathog. 2020 Dec 3;16(12):e1009055. doi: 10.1371/journal.ppat.1009055 (PMC7714144; doi:10.1371/journal.ppat.1009055)

Figure S1

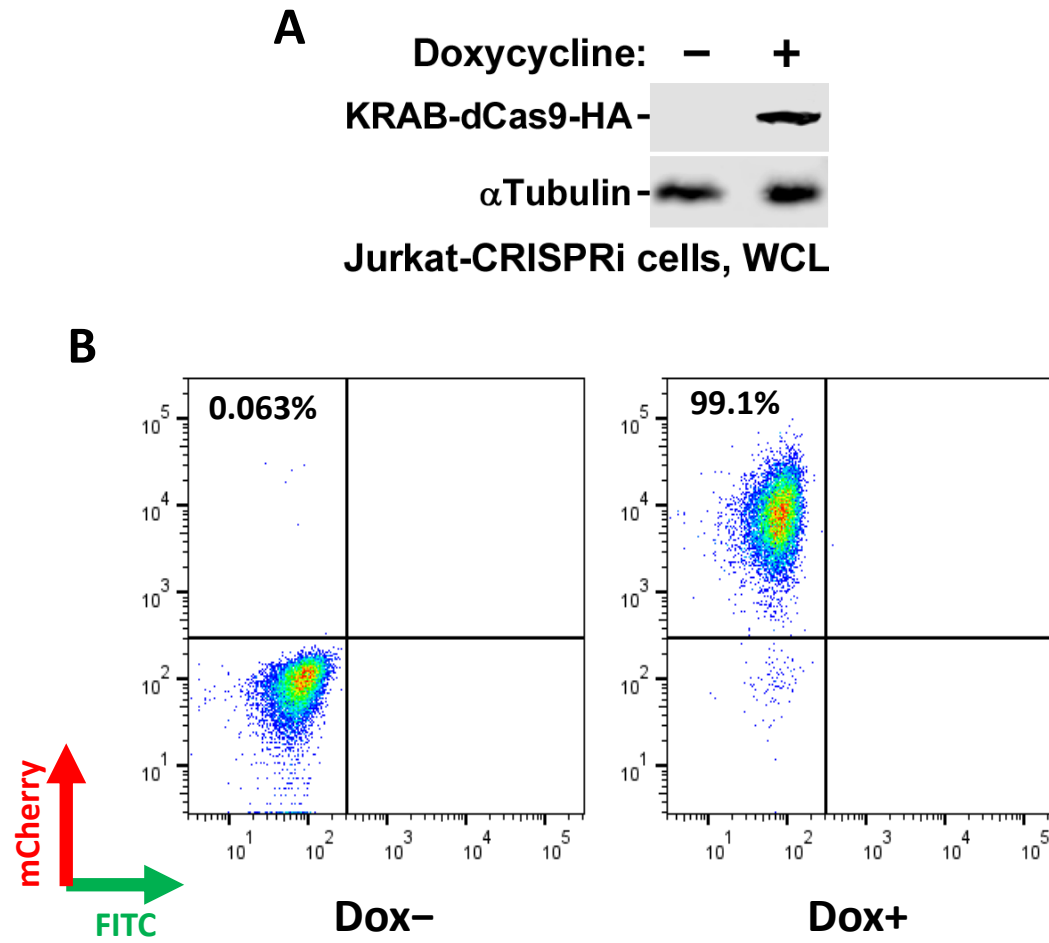

Supplement: S1 Fig — A. Results of Western blot analyses of the Dox-inducible expression of the KRAB-dCas9-HA fusion protein in the whole cell lysates (WCL) of Jurkat-CRISPRi cells. B. Representative FACS plots showing the Dox-inducible expression of the mCherry fluorescent protein as a reporter for the expression of KRAB-dCas9-HA fusion protein in the Jurkat-CRISPRi cells. In both panels, the cells were treated with either 0.1% DMSO (CRISPRi−) or 1 μg/ml Dox (CRISPRi+) for 2 days. (PDF) [file ppat.1009055.s001.pdf]

Figure S2

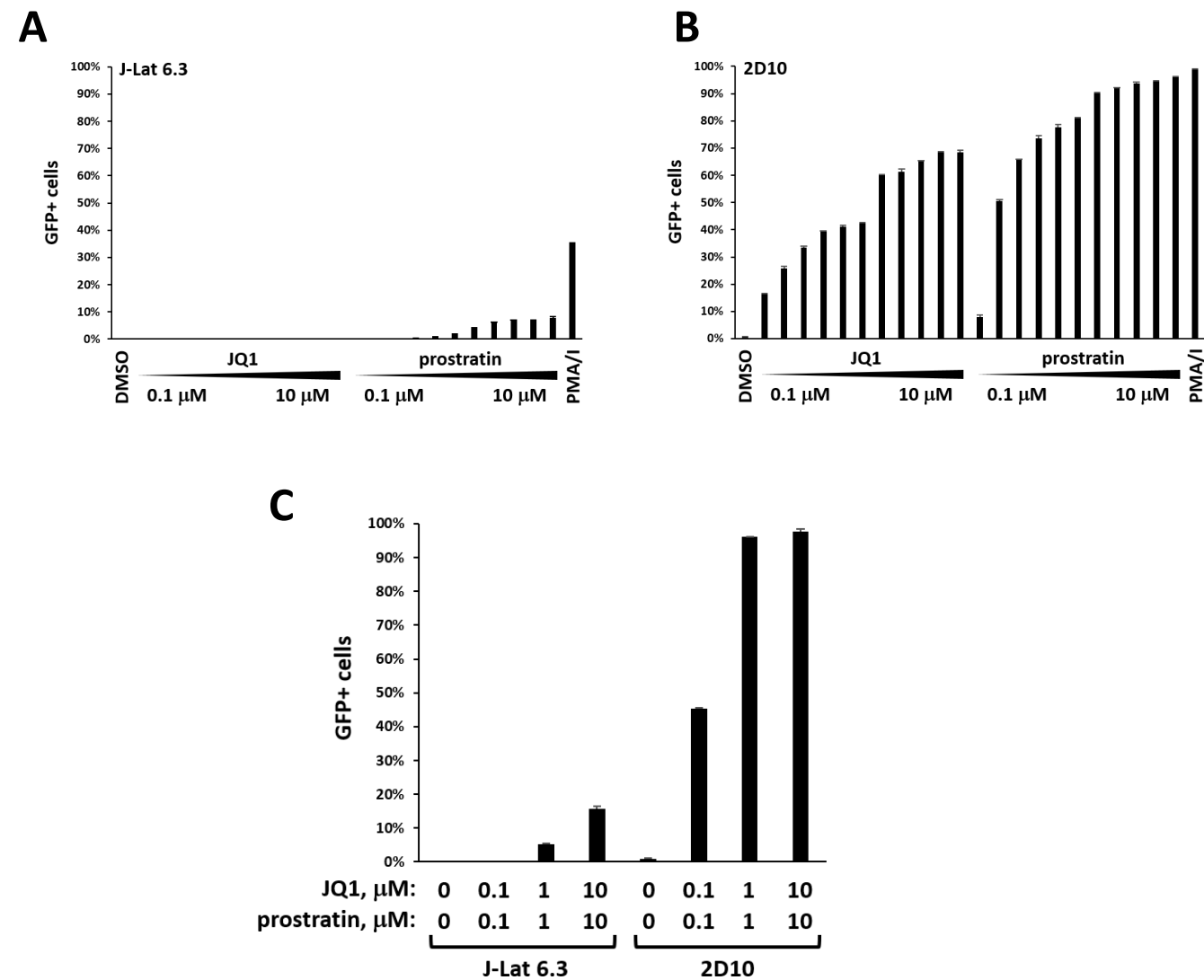

Supplement: S2 Fig — A., B., & C. The J-Lat 6.3 and 2D10 cell lines were treated with 0.1% DMSO, varying concentrations (0.1 μM, 0.2 μM, 0.4 μM, 0.6 μM, 0.8 μM, 1 μM, 2 μM, 4 μM, 6 μM, 8 μM, 10 μM) of JQ1 or prostratin, combinations of the two drugs, or 50 ng/ml (81 nM) PMA and 1 μM Ionomycin for 20 hours. The treated cells were then subjected to FACS analyses to determine the percentages of GFP+ cells in each population. Error bars represent mean +/− standard deviation (SD) from three experimental replicates. (PDF) [file ppat.1009055.s002.pdf]

Figure S3

CRISPRi- CRISPRi+

**A**

JiL-1 cells:

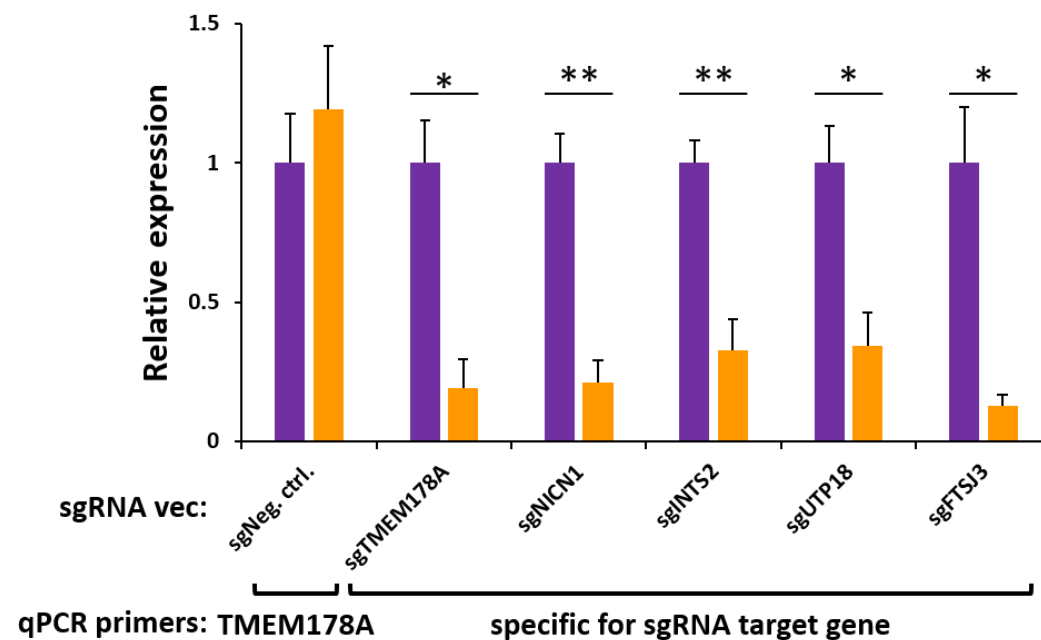

**B**

JiL-1 cells:

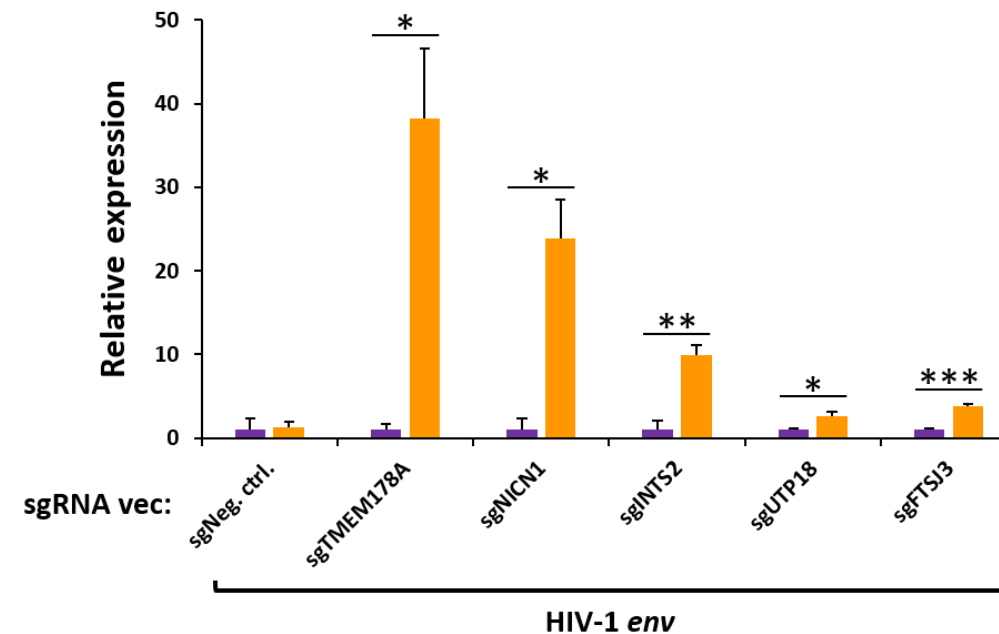

**C**

JiL-2 cells:

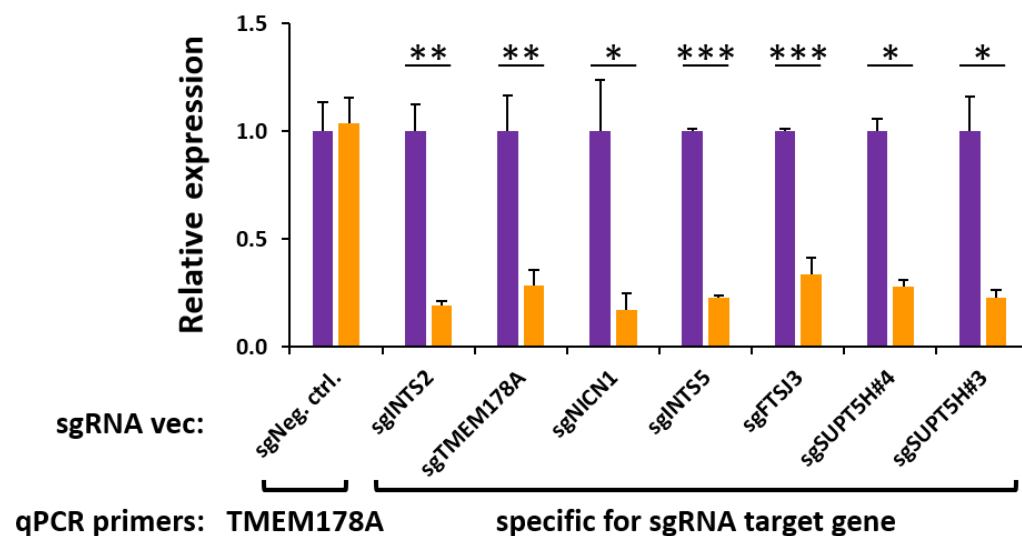

**D**

JiL-3 cells:

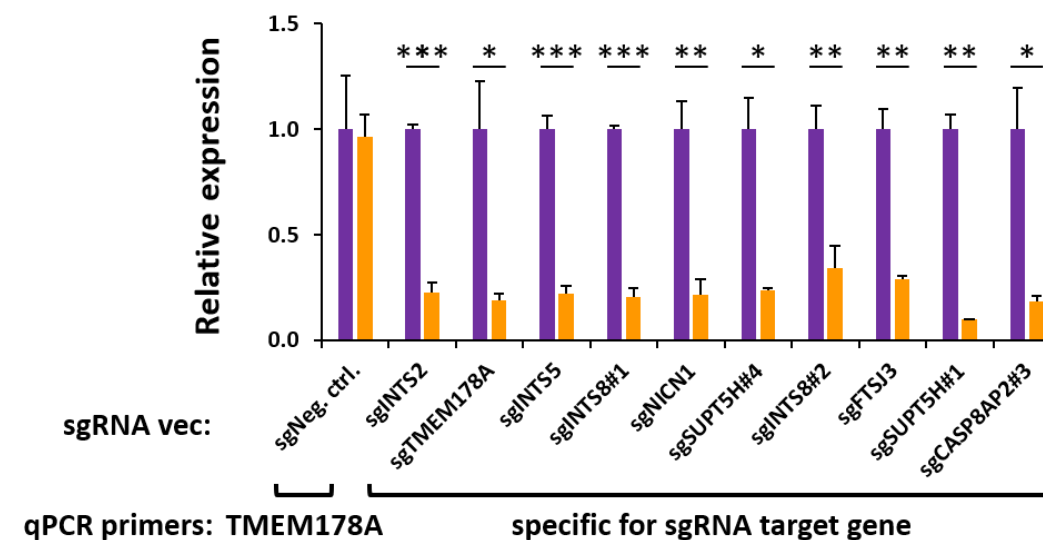

Supplement: S3 Fig — A., B., C. & D. RT-qPCR analyses of the mRNA levels of the genes that are denoted by the corresponding qPCR primers. The JiL cells were first transduced with the indicated sgRNA vectors, selected in the presence of puromycin, and then treated with either 0.1% DMSO (CRISPRi−) or 1 μg/ml Dox (CRISPRi+) for 3 days. The JiL-1 cells were also treated by 1 μM JQ1 + 0.2 μM prostratin for 20 hours before analyses. The mRNA levels detected in the CRISPRi− cells were set to 1. Error bars represent mean +/− SD from three experimental replicates. Asterisks denote levels of statistical significance calculated by two-tailed Student’s t-test (*: p<0.05, **: p<0.01, and ***: p<0.001). (PDF) [file ppat.1009055.s003.pdf]

Figure S4

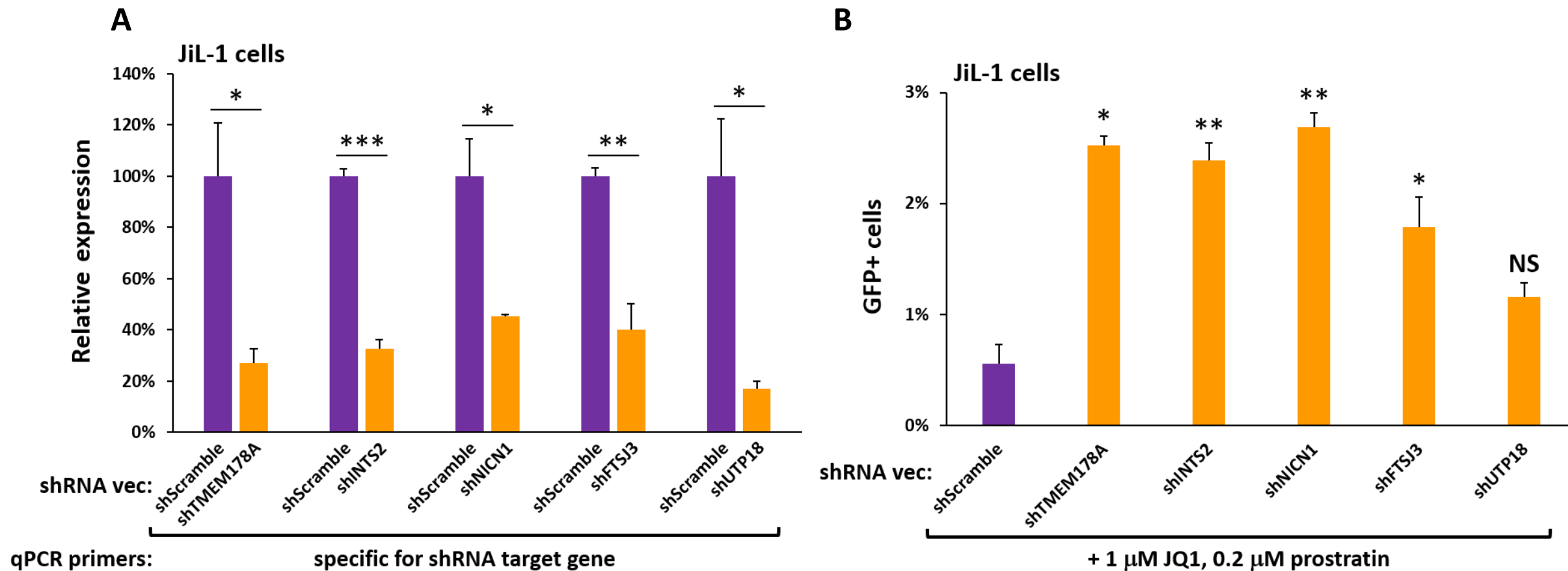

Supplement: S4 Fig — The JiL-1 cells were first transduced with the indicated shRNA vectors, and then selected by 1 μg/ml puromycin for 3 days. A. The cells were then subjected to RT-qPCR analyses of the mRNA levels of the genes that are denoted by their corresponding qPCR primers. B. All the cells were treated by 1 μM JQ1 + 0.2 μM prostratin for 20 hours, and then examined by flow cytometry for GFP+%. In both panels, error bars represent mean +/− SD from three experimental replicates. Asterisks denote levels of statistical significance calculated by two-tailed Student’s t-test (*: p<0.05, **: p<0.01, and ***: p<0.001). (PDF) [file ppat.1009055.s004.pdf]

Figure S5

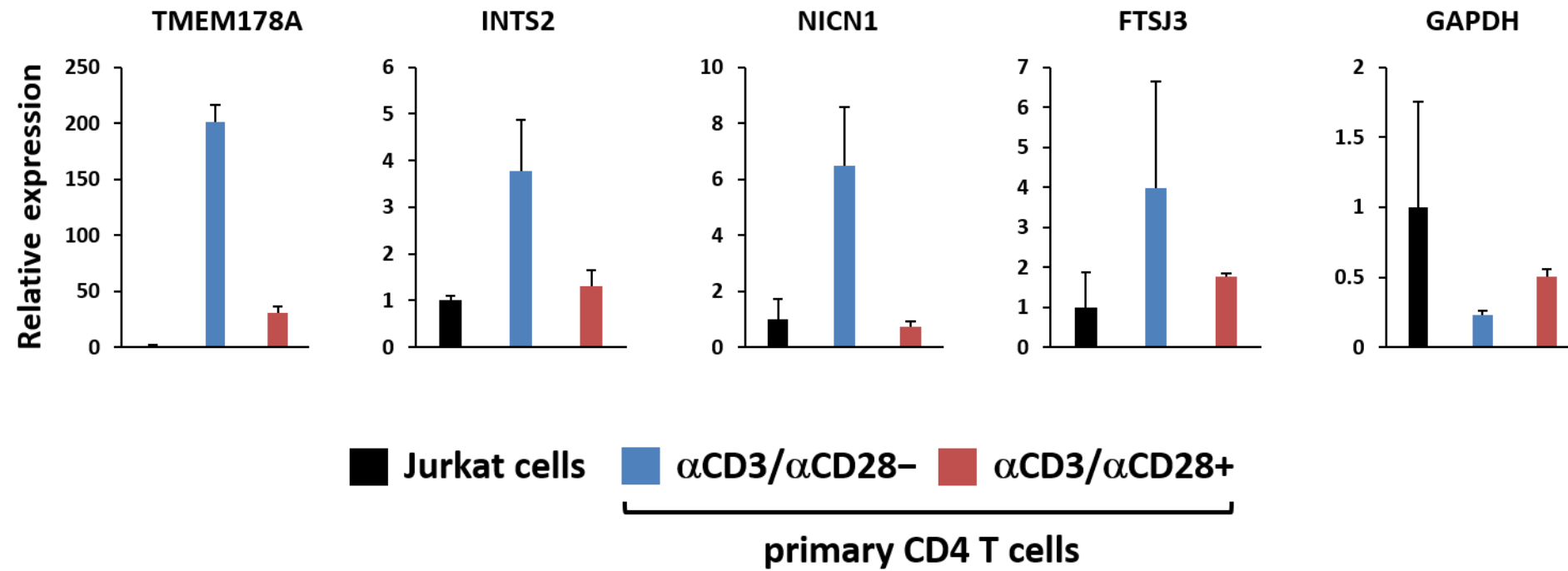

Supplement: S5 Fig — RT-qPCR analyses of the mRNA levels of the indicated genes in Jurkat cells and primary CD4 T cells before and after activation by antibodies against CD3 and CD28. To allow comparison, the mRNA level detected in the Jurkat cells was set to 1. Error bars represent mean +/− SD from three experimental replicates. (PDF) [file ppat.1009055.s005.pdf]
