# Supplementary material for: Identification of unrecognized host factors promoting HIV-1 latency
Source: PLoS Pathog. 2020 Dec 3;16(12):e1009055. doi: 10.1371/journal.ppat.1009055 (PMC7714144; doi:10.1371/journal.ppat.1009055)
Supplement: S4 Table — (DOCX) [file ppat.1009055.s009.docx]

**Table S2. List of DNA oligoes used in this study**

| Oligoes | Sequence (5’ 🡪 3’) |
| --- | --- |
| For shRNA cloning: | |
| shTMEM178A-FWD | CCGGTTCTTCTTTCTCTACACCTCTCGAGAGGTGTAGAGAAAGAAGAATTTTTG |
| shTMEM178A-REV | AATTCAAAAATTCTTCTTTCTCTACACCTCTCGAGAGGTGTAGAGAAAGAAGAA |
| shINTS2-FWD | CCGGTTGTATTATTCTGGGAAGGCTCGAGCCTTCCCAGAATAATACAATTTTTG |
| shINTS2-REV | AATTCAAAAATTGTATTATTCTGGGAAGGCTCGAGCCTTCCCAGAATAATACAA |
| shNICN1-FWD | CCGGTTTCCTGCAACTCGAAGGGCTCGAGCCCTTCGAGTTGCAGGAAATTTTTG |
| shNICN1-REV | AATTCAAAAATTTCCTGCAACTCGAAGGGCTCGAGCCCTTCGAGTTGCAGGAAA |
| shFTSJ3-FWD | CCGGTATCTATGAGGTCTCTCTTCTCGAGAAGAGAGACCTCATAGATATTTTTG |
| shFTSJ3-REV | AATTCAAAAATATCTATGAGGTCTCTCTTCTCGAGAAGAGAGACCTCATAGATA |
| shUTP18-FWD | CCGGAGACATAAAGAACCTTGCTCTCGAGAGCAAGGTTCTTTATGTCTTTTTTG |
| shUTP18-REV | AATTCAAAAAAGACATAAAGAACCTTGCTCTCGAGAGCAAGGTTCTTTATGTCT |
| shScramble-FWD | CCGGCCTAAGGTTAAGTCGCCCTCTCGAGAGGGCGACTTAACCTTAGGTTTTTG |
| shScramble-REV | AATTCAAAAACCTAAGGTTAAGTCGCCCTCTCGAGAGGGCGACTTAACCTTAGG |
| For RT-qPCR: | |
| qActB-F | AGAGCTACGAGCTGCCTGAC |
| qActB-R | AGCACTGTGTTGGCGTACAG |
| qGAPDH-F | AATCCCATCACCATCTTCCAG |
| qGAPDH-R | AAATGAGCCCCAGCCTTC |
| qEnv-F | GAGACAGAGACAGATCCATTCG |
| qEnv-R | CCAGAAGTTCCACAATCCTCG |
| qGFP-F | AAGCTGACCCTGAAGTTCATCTGC |
| qGFP-R | CTTGTAGTTGCCGTCGTCCTTGAA |
| 5' LTR-gag-F | GCCTCAATAAAGCTTGCCTTGA |
| 5' LTR-gag-R | GGGCGCCACTGCTAGAGA |
| qTMEM178A-F | CTGGACTCCTGTTCCTCATG |
| qTMEM178A-R | AGCCGGTTCAAATCATACGAG |
| qFTSJ3-F | ACAGAACGTTGTAGGCAGG |
| qFTSJ3-R | AAACGTAGAGCCATCAGTGTC |
| qNICN1-F | CAGTCACACCTCCGCAAG |
| qNICN1-R | GTGTAGGAGAGCAAGTTCAGG |
| qUTP18-F | TCCACATCAACTTCTCTTCCAAG |
| qUTP18-R | CTAATCCAGCAACCATCACAATC |
| qINTS2-F | AATGAATCCTTCTCAGGCCC |
| qINTS2-R | CTCACTCCATCCTCACAAGC |
| qCD25-F | GCTGTGTTTTCCTGCTGATC |
| qCD25-R | ACTTCTGTTGTCTGTTCCCG |
| qCD69-F | ACATGGTGCTACTCTTGCTG |
| qCD69-R | CTTTGCCATTTGACCACTTCC |
| \| qPSMD3-F \| \| --- \| \| qPSMD3-R \| \| qNFKBIA-F \| \| qNFKBIA-F \| \| qMINA-F \| \| qMINA-R \| \| qUCHL5-F \| \| qUCHL5-R \| \| qINTS5-F \| \| qINTS5-R \| \| qSUPT5H-F \| \| qSUPT5H-R \| \| qINTS8-F \| \| qINTS8-R \| \| qCASP8AP2-F \| \| qCASP8AP2-R \| \| qLTR-TSS-F \| \| qLTR-190nt-R \| | \| CGCCTCAACCACTATGTTCTG \| \| --- \| \| AATCAGCCTCTGTGTCCATG \| \| GTCTACACTTAGCCTCTATCCATG \| \| AGGTCAGGATTTTGCAGGTC \| \| GCACCAAAGAACTGCTTTCC \| \| CGGCAGTACTGTGAGGACAA \| \| TGGTTCAGGACTCCCGACTT \| \| CTGGTGGGTACAGTTCAGTAAC \| \| CAGTTATATGCAGGGCTAGTGG \| \| GTGAAGGGAGGCTGTAATGTC \| \| ACTGGGCGAGTATTACATGAAG \| \| ACTGGGCGAGTATTACATGAAG \| \| AGGAGTCACTGTTGGAGAAAC \| \| TTGTTATCAGGCGGAGGTTG \| \| GATGACAATGGTGATGGAACAAG \| \| TGGTACACAGGATTTGGAAGC \| \| GGGTCTCTCTGGTTAGACCAG \| \| CTGCTAGAGATTTTCCACACTGAC \| |
| For ChIP-qPCR: | |
| \| HIV-nuc1-F \| \| --- \| \| HIV-nuc1-R \| \| HIV-env-F \| \| HIV-env-R \| | \| CTGGGAGCTCTCTGGCTAACTA \| \| --- \| \| TTACCAGAGTCACACAACAGACG \| \| TGAGGGACAATTGGAGAAGTGA \| \| TCTGCACCACTCTTCTCTTTGC \| |
